# Process evaluation of a pragmatic, multicentre pilot Randomised Controlled Trial (RCT) in primary care: Tailored intervention for COPD and Co-morbidities by Pharmacists and Consultant Physicians (TICC PCP)

**DOI:** 10.1371/journal.pone.0326178

**Published:** 2025-06-30

**Authors:** Karen Wood, Richard Lowrie, Georgia Smith, David Anderson, Jane Moir, Lynda Attwood, Andrew McPherson, Aziz Sheikh, Elaine Rankine, Frances S. Mair

**Affiliations:** 1 School of Health and Wellbeing, College of Medical, Veterinary and Life Sciences, The University of Glasgow, Scotland, United Kingdom; 2 Centre for Homelessness and Inclusion Health, School of Health in Social Science, The University of Edinburgh, Scotland, United Kingdom; 3 Pharmacy Services, NHS Greater Glasgow and Clyde, Scotland, United Kingdom; 4 NHS Greater Glasgow and Clyde, Scotland, United Kingdom; 5 NHS Lothian, Scotland, United Kingdom; 6 Centre for Cardiovascular Sciences, University of Edinburgh, Scotland, United Kingdom; 7 Usher Institute, College of Medicine and Veterinary Medicine, The University of Edinburgh, Scotland, United Kingdom; 8 Nuffield Department of Primary Care Health Sciences, University of Oxford, Oxford, United Kingdom; Torrens University Australia, AUSTRALIA

## Abstract

Chronic Obstructive Pulmonary Disease (COPD) is a growing global challenge. We undertook a process evaluation embedded within the Tailored Intervention at home for patients with moderate-to-severe COPD and Co-Morbidities by Pharmacists and Consultant Physicians (TICC PCP) pilot randomised controlled trial (RCT), which explored patient/stakeholder perceptions of the intervention, acceptability of trial procedures, and barriers/facilitators to intervention implementation. Semi-structured telephone interviews were conducted with intervention patients (20) and stakeholders (10); data were analysed thematically, conceptualised through Normalisation Process Theory. Patient perspectives compared based on socio-economic status (SES). Patients/stakeholders reported positive perceptions of the intervention/trial procedures. Pharmacists provided support across a range of health/social issues. Challenges related to: recruitment; workload/lone-working; managing patient complexity; and data collection. There were suggestions Pharmacists were able to undertake more actions to support patients from low SES areas. Overall, intervention and trial procedures were acceptable to patients and stakeholders. Findings support progression to full-scale RCT.

## Introduction

Chronic Obstructive Pulmonary Disease (COPD) prevalence is increasing globally [1], presents a major burden to healthcare systems [2] and is one of the top four leading causes of Disability-Adjusted Life Years (DALYs) among people aged over 50 [3]. There is a clear association between socioeconomic status (SES) and COPD prevalence [4,5] with the incidence and prevalence of COPD in the most deprived quintile estimated to be at least double that of the least deprived in Scotland [4,6]. Low SES is associated with increased likelihood of hospitalisation due to COPD [5].

A feasibility study (on which the pilot trial in this paper was based) suggested home-based collaborative Pharmacist and Consultant Respiratory Physician interventions may provide a complementary approach to community care, which could reduce the risk of key COPD-related adverse outcomes (e.g. exacerbations and hospitalisations) [7]. Two UK pilot studies [7,8] and an Australian interdisciplinary intervention for COPD, which included a Pharmacist home-medicines-review [9], reported moderate improvements in key COPD outcomes. However, the studies had limited generalisability – for example, due to small sample size and short follow up [8]. The interventions were not delivered by Pharmacists who had received training to prescribe independently or review and treat respiratory and other co-morbidities. Furthermore, patient and healthcare professional (HCP) perceptions of the interventions were not formally evaluated, although informal patient feedback was positive [7–9]. Patients appreciated the time taken by Pharmacists, found the face-to-face contact valuable and improved their inhaler technique. However, lack of a perceived need for a home medicines review contributed to poor uptake of one intervention [9].

In accordance with stages of testing novel complex interventions and following a successful feasibility study [7], the Tailored Intervention at home for patients with moderate-to-severe COPD and Co-Morbidities by Pharmacists and Consultant Physicians (TICC PCP) pilot randomised controlled trial (RCT) was undertaken in Scotland. The intervention involved home visits by Pharmacist Independent Prescribers working collaboratively with Consultant Respiratory Physicians over a one-year period, with the aim of improving the management of COPD and other comorbidities by providing holistic care. The additional qualifications attained by Pharmacist Independent Prescribers in the UK, allow them to undertake clinical evaluations and prescribe any medicines within their competence. In the context of the TICC PCP intervention, this enabled Pharmacists to independently visit participants in their homes, carry out assessments and prescribe/deprescribe medication in relation to their COPD and other health conditions. This model has been utilised in other targeted groups [10]. The TICC PCP intervention was provided in addition to care as usual. The control group continued to receive care as usual.

Understanding patient perceptions is essential when evaluating a complex intervention [11] as acceptability influences patient adherence and future implementation [12]. Stark social patterning in COPD may interact with the intervention and any potential influence on health inequalities [5,12].

We conducted a process evaluation embedded within the pilot RCT. This study aimed to: (1) explore patient and stakeholder perceptions of the TICC PCP intervention (how it was understood, engagement between patients and Pharmacists and others involved in delivering the intervention, how useful the intervention was) and trial, including the acceptability of trial procedures; and (2) identify likely barriers and facilitators to future implementation of the intervention.

## Methods

### Ethics

All patients and stakeholders gave informed, written consent at enrolment and the TICC PCP pilot RCT was approved by the South-East Scotland Research Ethics Committee (20/SS/0093) and is registered with the ISRCTN Registry [https://www.isrctn.com/ISRCTN43508703].

### TICC PCP intervention

The intervention was provided by National Health Service (NHS) Pharmacist Independent Prescribers collaborating with Consultant Respiratory Physicians employed by two health boards in Scotland (NHS Greater Glasgow and Clyde and NHS Lothian), in addition to usual care. All participants continued to receive usual care throughout the study – usual care is outlined here. In Scotland, NHS primary and secondary health care is free at the point of use, including prescriptions which are dispensed (filled) in community pharmacies. Patients are registered with one General Practitioner (GP) (Family Physician) where they receive primary health care for non-emergency acute and chronic conditions. GPs co-ordinate comprehensive care and referrals to secondary care based respiratory physicians (including specialist COPD clinics) as needed. Complete clinical records (primary care and hospital consultation notes, prescribing, referrals, laboratory results etc.) are held by GP practices. At recruitment, all participants were under the care of Consultant Respiratory Physicians, after having been referred to local hospital based respiratory clinics. Participants were expected to attend the hospital clinics to receive care and to attend their GP surgery to receive care for COPD and any other health problem.

Independent Prescribers have successfully undertaken an independent prescribing qualification, enabling Pharmacists in the UK to prescribe any medicine. The intervention was tailored to patients’ needs as assessed by the pharmacists. Typically, Pharmacists visited intervention patients repeatedly at home over a one-year period, monthly during the first six months and every second month in the second six months (Pharmacists worked two days per week for the first six months, then one day per week for the next six months). Pharmacists undertook initial holistic assessments of patients’ COPD symptoms and management (including medication), comorbidities and social circumstances (e.g. housing, benefits). Working in conjunction with patients’ Respiratory Consultants and GPs, Pharmacists adjusted or deprescribed existing or prescribed new medications for COPD and comorbidities and referred to other services. Where appropriate, Pharmacists conducted or referred patients for additional tests, to make new diagnoses. Pharmacists updated medical records and informed other HCPs of treatment changes or issues. Intervention (n = 53) and control participants (n = 55) were visited by Researchers to complete study questionnaires and physical measures 3-monthly for 21 months.

### Process evaluation

#### Sampling and recruitment.

Intervention patients were purposively sampled according to SES, sex and intervention site. Participants from the control (care as usual) group were not included in the process evaluation. Using baseline trial data, patients were assigned to quintiles of deprivation using the Scottish Index of Multiple Deprivation (SIMD), an area based measure of SES [13]. Patients from the 1st and 2nd most deprived quintiles were defined as ‘low SES’, those in the 4th and 5th least deprived quintiles as ‘high SES’, while those in the 3rd quintile were defined as ‘mid SES’.

Patients were interviewed between three- and eleven-months post RCT recruitment, thus ensuring experience of pharmacist input. Qualitative researchers (GS, KW) checked patients were well enough to participate prior to contacting them. Patients were then called within a week, reminded what interviews would entail and verbally reconsented before interviews. Four intervention patients (all low SES) declined to participate in an interview: two did not want to complete an interview, one felt unable to speak for long periods, and one did not want to complete a telephone interview. A further patient initially agreed to participate, but scheduling attempts were unsuccessful.

Stakeholders were purposively sampled to include those involved in delivering the intervention and RCT (Pharmacists – two; Respiratory Consultants – two; Researchers – three; and Administrators – one) or whose patients were involved in the trial (one GP, external to study team) and were interviewed along with one additional external Respiratory Consultant identified through team contacts. Interviewing stakeholders external to the study team provided additional insight from those not closely involved in delivering the intervention and trial. Only two of those invited to participate were unable to – one external Respiratory Consultant felt they were not best suited to being interviewed, and one GP did not have the time available.

### Data collection

Semi-structured telephone interviews (chosen in part due to the ongoing COVID-19 pandemic) lasting around one hour were completed between December 2021 and November 2022 (KW, GS) to explore patients’ and stakeholders’ perceptions of the intervention using separate interview guides informed by the concepts of Normalization Process Theory (NPT) (S1 File). NPT is used to explore the extent to which practices become routinely embedded in everyday life [14] and has been used extensively to evaluate complex interventions and self-management of chronic conditions [15]. From the perspective of NPT, four kinds of work are involved in a practice becoming embedded: coherence (sense making work); cognitive participation (relational work); collective action (operational work); and reflexive monitoring (appraisal of the practice) [14]. The interview schedules (S1 File) were further developed iteratively based on initial interviews, e.g. to include questions about effects of the COVID-19 pandemic on care.

Both interviewers were female: one an experienced qualitative researcher, the other a medical student without prior qualitative data collection experience, who was guided and supervised by the former. Interviewers did not have prior research experience in relation to COPD. They had no prior relationships with patient participants, but some stakeholders were known to them. Due to breathing difficulties experienced by patients and to facilitate participation, where one hour was felt to be too long for patients to speak, they were offered the option to complete the interview in two parts. Short breaks were also offered where necessary. Family members or carers were occasionally present at the time of the interview. Interviews were digitally audio recorded, transcribed verbatim and then checked (GS and KW). Transcripts were not shared with participants.

### Data analysis

Inductive thematic analysis was used to derive themes and data saturation was achieved [16]. Coding was supported by NVivo 10 software and carried out using codebooks developed from initial coding of interviews. Eight patient interviews were double coded by two researchers (GS, KW). Themes were identified based on prevalence and relevance to research questions. Coding clinics were utilised (GS, KW, FSM) to review, sense check and refine coding and themes (S1 File). Themes were mapped where appropriate to NPT constructs to aid conceptualisation. Participants did not provide feedback on the findings. Themes are illustrated through use of quotations – to protect anonymity, quotes provided by study team and health professionals were attributed to e.g., ‘Stakeholder 1’ or ‘Stakeholder’, without further detail. Perspectives of high and low SES patients were compared.

## Results

Interviews were completed with 20 intervention patients and 10 stakeholders (HCPs/study team members) from both study sites (Tables 1 and 2).

**Table 1:** Interview patient characteristics.

Patient characteristics		N = 20
Sex	Male	10
	Female	10
Age	50-59	2
	60-69	8
	70-79	8
	80-89	2
Socio-Economic Status (SIMD* quintile)	1 (most deprived)	8
	2	3
	3	1
	4	2
	5 (least deprived)	6
Study site	Glasgow	11
	Lothian	9
Number of months in intervention at point of interview (months)	3	3^‡^
	4	3
	5	4
	6	3
	7	4
	8	2^‡^
	11	1^‡^
Modified MRC score – Mean (SD)		3.0 (1.2)
COPD Assessment Test – Mean (SD)		24.2 (9.1)
Exacerbation (rescue pack) in past 12 months (%)	More than 2 packs	12 (63.2%)
	2 packs or less	7 (36.8%)
	Data not available	1

*SIMD = Scottish Index of Multiple Deprivation.

‡ 1 patient in each of these groups started their interviews at this time point, however due to interruption or patients’ preference the interview was paused and a second part completed around 1 month later.

**Table 2:** Interview stakeholder characteristics.

Stakeholder characteristics		N = 10
Sex	Male	4
	Female	6
Study site	Glasgow	6
	Lothian	4
Profession	Pharmacist	2
	Researcher	3
	Administrator	1
	Respiratory Consultant	3
	General Practitioner	1

**Fig 1:**
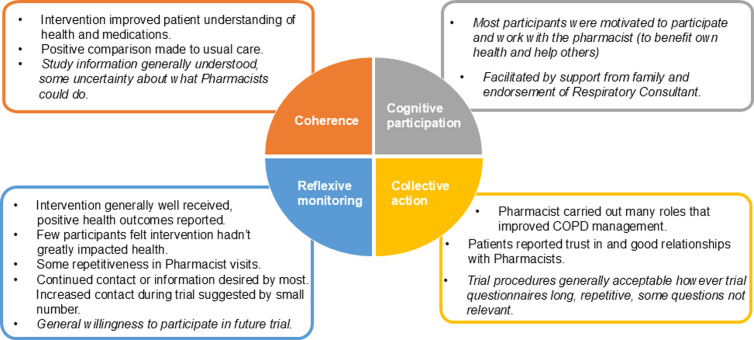
Normalisation Process Theory conceptualisation of patient perspectives. Main themes identified in patient data mapped to four constructs of Normalisation Process Theory (Coherence, Cognitive participation; Collective action; Reflexive monitoring). Includes patient perspectives on the intervention and in italics, patient acceptability of trial procedures.

**Fig 2:**
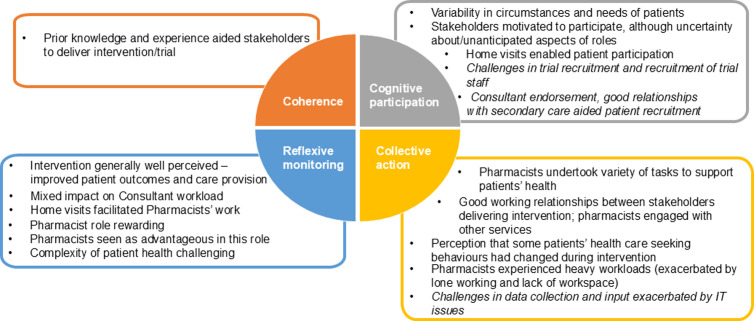
Normalisation Process Theory conceptualisation of stakeholder perspectives. Main themes identified in stakeholder data mapped to four constructs of Normalisation Process Theory (Coherence, Cognitive participation; Collective action; Reflexive monitoring). Includes stakeholder perspectives on the intervention and in italics, stakeholder acceptability of trial procedures.

Below we report patient perspectives of the intervention followed by stakeholder views (Fig 1, Fig 2). Details of barriers and facilitators to a future trial or implementation of the intervention are then presented.

### Patient perception of intervention

#### Patient understanding and perception of COPD and intervention (Coherence).

Most patients had limited understanding of COPD. Some described delayed, absent or poor-quality information giving about their condition. Deterioration in physical and psychological symptoms related to COPD was common, with limitations experienced in the completion of daily tasks, hobbies and employment (Table 3).

**Table 3:** Patient perception of intervention.

Patient perception of intervention
**Patient understanding and perception of COPD and intervention:**
*“…I was diagnosed back in, erm, 2012…But I didn’t find out till 2015. So in-between that time I kind of worked very hard at my body doing more. So, by 2015, I was pretty done in…see, I’m deaf, and so the doctor could have very well telt me, but if she’s facing the screen, then I’m not hearing what she’s saying properly.” Participant 1, Female, Low SES, Glasgow.*
**Impact of intervention on understanding of health:**
*“Then she gave me a booklet to help me with the diagnosis, to explain what was happening inside my body you know. So that was very helpful you know. You can read COPD up on the internet, but you get all these things that the more you read the worse it gets.” Participant 13, Male, High SES, Lothian.*
**Pharmacist compared to other HCPs**:
*“When you go to a doctor or anything like that, they just seem to throw tablets at you, but she’ll have a talk with you about it and it’s a lot more…and it’s easier to understand…It seems different.” Participant 2, Male, Low SES, Lothian.*
**Operationalisation of intervention**
**Relationship with Pharmacist:**
*“…you’re having a conversation, it’s like having a friend in to visit.” Participant 7, Male, Low SES, Lothian.*
**Evaluation of intervention**
**Positive impact:**
*“I thought it was quite good because when she comes out, she’ll sound my chest, take my blood pressure, my temperature, everything a doctor should be doing ‘cause they’ll not come to the house. So, she’s there doing it, and I think a lot of her for that. And she’s very nice.” Participant 2, Male, Low SES, Lothian.*
*“I think changing my medicine helped to ease my breathing and in general just talking to me about it…it did take a week or so, which I wasn’t expecting anything overnight, but it seems to ease my breathing a bit. It’s not as painful…” Participant 9, Male, High SES, Glasgow.*
**Limited impact on health:**
*“…I was quite impressed with [Pharmacist] and maybe somebody in a different situation…it was just unfortunate the way I am, nothing really made much of a difference.” Participant 17, Male, Low SES, Glasgow.*
*“I think it’s very similar to what the respiratory nurses give me…They’re not fighting each other with different advice; they’re very much on the same page.” Participant 19, Female, High SES, Lothian.*
**Repetitiveness in Pharmacist visits:**
*“It’s very awkward…when you’re talking about the same condition all the time, there’s only so much that you can talk about or [Pharmacist] can explain things to me… It’s just awkward, you’re just talking about the tablets or how I’m feeling, and there’s only so much that she can tell me, to help me, so it must be awkward for her as well.” Participant 9, Male, High SES, Glasgow.*

#### Patient understanding and perception of COPD and intervention – Impact of intervention on understanding of health (Coherence).

Many patients felt better informed about their medications after the intervention, in particular their purpose, mechanism, administration and reduction of side effects. Pharmacists also contributed to an improved understanding of COPD among a few participants (Table 3).

#### Patient understanding and perception of COPD and intervention – Pharmacist compared to other HCPs (Coherence).

Most patients reported limited previous interaction with Pharmacists, but felt study Pharmacists offered more accessible, regular contact, provided more information and explanation, and spent more time with them than other HCPs (Table 3).

#### Operationalisation of intervention – Pharmacist actions (Collective Action).

Across a variety of tasks (Table 4) Pharmacists offered information and expertise, reassurance and support, problem solving, bridged gaps in and between care and facilitated access and receipt of care and services for all patients*.*

**Table 4:** Pharmacist actions, patient and stakeholder perceptions.

Health area	Pharmacist action (Patient perspective)	Socio-Economic Status (SES) comparison	Participant quote	Pharmacist action (Stakeholder perspective)	Stakeholder quote
Regular health care professional contact and monitoring	Monitoring and assessment: blood pressure, chest examination, weight etc.		*“…I eventually had to go and take blood pressure tablets because the Pharmacist actually noticed my blood pressure was high. And then I went to the doctor’s and done a seven-day test, and that’s when they found out my blood pressure was high. So, it was through seeing the pharmacist that I got the blood pressure checked…because I would never have thought of going to the doctor with it.” Participant 14, Male, Low SES, Lothian*	Regular observations and health record checks	*“…because I referred somebody for a six-minute walking test and then because the result of that test showed she needed oxygen. So that then triggered a referral for an oxygen assessment.”*
	Send for further tests and scans on basis of encounters or review of health records.	Low SES patients in particular.		Referral for further investigations e.g. DEXA scans, blood tests	
	Provided information around test results.		*“…it was her that actually found out that I had diabetes and high cholesterol…She just wanted a blood test done…And it all came back in the bloods…But when I phoned the surgery, they just told me everything was alright…and it was [Pharmacist] that phoned them, and she got it sorted.” Participant 20, Female, High SES, Lothian *		
	Followed up patients after hospital discharge.	Reported by two low SES patients.			
Lifestyle	Smoking cessation advice		*“She has told me not to drink as much because I like a beer. She told me at least a few days through the week try not to drink, which I am doing.” Participant 13, Male, High SES, Lothian*	Smoking cessation advice	*“And trying to support them with other issues, so we’ve had addiction issues, not just cigarettes, there has been alcohol issues as well.”*
	Advice around nutrition/diete.g. managing difficulties with weight			Dietary advice and referral to dieticians	
			*“She’s always saying about, like, trying to do things. But because of my shaking and my nebuliser, it can make it a bit worse. And my anxiety, I find it difficult to do things that I used to do, like make jigsaws and sew and stuff like that. So, we’re looking at ways that we can do something about that. So, she got me a jigsaw, so we’re on it [laughs] aye.” Participant 1, Female, Low SES, Glasgow*		
	Alcohol consumption advice			Alcohol consumption advice	
	Exercise and physical activity – check on activity levels; advice.				
	Advice and support for other activities such as hobbies.				
Breathing/exacerbation management	Breathing techniques and exercises		*“She said…not to push myself too hard. She recognised in me that I push myself too hard; I don’t pace myself properly…But just recently, since I’ve been mobile again, I’ve learned to pace myself. So, I’ve been heeding her words.” Participant 8, Male, High SES, Lothian*	Determine causes of (frequent) exacerbations	*“…I’m trying to equip people with the tools to help them manage their COPD. I mean, it is as much as they can because some people are very, very poorly and there is only so much you can do. But I’ve certainly been working with [Respiratory Consultant] to identify patients who are having frequent exacerbations of COPD, having a look at why they are exacerbating and trying to work out strategies to try and minimise these exacerbations which can lead to hospital admission.”*
	Advice on using rescue packs/taking antibiotics			Strategies to reduce hospitalisations, further deterioration.	
	Referral to pulmonary rehabilitation	Two high SES patients	*“Well, because it’s getting picked up easier, when I’m not well, and I’m not getting pure ill before we realised I’m not well, we’re picking up on it sooner, so it’s not escalating the way it used to.” Participant 1, Female, Low SES, Glasgow*		
				Improve quality of life	
Medication review and change (Deprescribe, new medications, changes to existing medications)	Review existing medications.		*“…it kind of went for a while without me taking the tablet, and nobody knowing that I wasn’t taking the tablets. So, once she picked up on that, she was right on it, and got that sorted, so that I got the appointment.” Participant 1, Female, Low SES, Glasgow*	Reviewed existing medications. Prescribed new or made changes to, existing medications.	*“So, I suppose I do a sort of polypharmacy review on the patient when I visit their home.”*
	Identify medications no longer required or not being taken.		*“She knew all my medication that I was on, you know. She was explaining to me different ones, and this is for that, whatever, you know, what do you think? Do you think you could do without that?” Participant 18, Female, Low SES, Glasgow*	Referrals for long-term antibiotics	*“…trying to identify patients for long-term antibiotic therapy to prevent exacerbation.”*
	Provided information and explanation around medications		*“Funnily enough, she also picked up on that I wasn’t actually holding one of the inhalers the correct way…which I hadn’t actually thought of. This is what I mean, she sits and watches you, you know, and she said, turn it around.” Participant 19, Female, High SES, Lothian*	Provided rescue packs.	*“Rescue packs, quite a lot of them we’ve given rescue packs to…didn’t have them before.”*
	Ensure medications being taken correctly.	Two high SES patients assisted with inhaler technique.	*“And she gave me a prescription for a Beclomethasone spray, because I’d said to her, the problem when I do have a cough is I find it hard to breathe…And she says, right, we’ll try you on this, and I thought, this is the best thing I’ve ever had.” Participant 17, Male, Low SES, Glasgow*	Referred/made changes to oxygen therapy	*“But I have learned people who might benefit from different types of oxygen, not just continuous oxygen like ambulatory oxygen. Where it would maybe desaturate when they mobilise.”*
	New medications (e.g. daily antibiotic for COPD; inhalers, supplements)	Both high and low SES patients received new medications or had medication changes. Multiple new medications or medication changes, more common among low SES patients.	*“…I was breathless and then she changed the inhalers. And I’ve got to admit, I feel a difference with the inhalers…But I felt they did help me a lot better than the ones I was on before.” Participant 4, Female, Low SES, Glasgow*		
	Existing medications changes, including dosages.				
Medication (prescriptions)	Provided prescriptions (expediting process, removing need to contact GP practices).		“*And that’s one thing [Pharmacist] always asked when she came to visit was, have you got your emergency supply of antibiotics and steroids. So, she wrote me out a prescription for that.” Participant 16, Female, Low SES, Glasgow*		*“And I email the GP who is the usual care GP for that patient to let them know if I’ve issued a prescription…”*
	Resolved prescription problems (e.g. delays, incorrect medicines, resistance from GPs)	Mainly low SES patients.			
	Provided and/or replaced rescue packs	Six patients (four low SES) described receiving rescue pack for first time.	*“But if I need any medication, like she got me, you’re supposed to have a rescue pack, which is antibiotics and steroids in the house. And so, I didn’t have any, and my doctor didn’t supply that.” Participant 3, Female, Low SES, Glasgow*		
Mental health	Opportunity to share worries and concerns.	Mainly mentioned by high SES patients.	*“She’s left me a tablet to take is I feel agitated or cannae get to sleep…which I’ve been taking…” Participant 2, Male, Low SES, Lothian*	Prescribed medications.	*“Maybe we’ve managed to help patients who had anxiety issues slightly, just with some medicines. I wouldn’t say we’ve done a huge amount of anxiety management holistically…I suppose we’ve maybe been able to signpost them for some apps with mindfulness and looking at different, various websites…”*
	Referred/asked patients to self-refer to mental health teams and counsellors.	More high SES patients.	*“The main thing’s the mental side of it…without a doubt, she was the catalyst for me getting help, mentally.” Participant 8, Male, High SES, Lothian*	Referred/asked patients to self-refer to mental health services.	
				Engaged with Respiratory Consultant and GP to support severe mental health problems.	
	Medication changes.		*“…she came in to see me, and she took one look at me, she says, you’re not right. She actually went and spoke to the GP, and she thought I should speak to a counsellor. She pinpointed that ‘cause she came in. I wouldn’t have phoned the GP and said I need to speak to a counsellor, or I need help. I wouldn’t have done that. It’s just not in my nature.” Participant 19, Female, High SES, Lothian*	Support for those experiencing bereavement and loneliness/social isolatione.g. Link Workers.	*“…I managed to refer somebody who has then had some physiotherapy and some occupational therapy, so they’ve had anxiety management through the occupational therapist…”*


Housing/home adaptations and mobility aids	Referred or organised delivery and installation of mobility aids and home adaptations (wheelchairs, walkers, seats, handrails, bannisters, rails/seats in showers, intercom system, fall alarm)	Referred to more often by low SES patients	*“…she did things like getting the social worker or OT, whatever…you know for the stuff I need. You know, she’s been really helpful. It’s been really helpful. I would never have done any of that…that banister, I wouldn’t have known.” Participant 4, Female, Low SES, Glasgow*	Organising adaptations and aides	*“So, we’ve done quite a lot of referrals to occupational health or therapy and we’ve managed to get things like bannisters and perching stools and anterior walkers to allow them to maybe be a wee bit more mobile.”*
				Referral to e.g. Occupational Therapy	
			*“And she’s gave me a walking stroller, with feet on it, so I can sit down when I need to, now. And a side bit for my bed.” Participant 20, Female, High SES, Lothian*	Identified housing circumstances detrimental to health (in conjunction with Respiratory Consultant, a patient was rehoused)	
			*“’Cause she was asking me all the different questions so that…to see if there was any other aids that she could actually get for me…the one thing that the two of us agreed on…it was standing doing my dishes…doing my ironing…can make me very breathless. And that’s what she says, well we can get you a chair…and it does help. It definitely does.” Participant 16, Female, Low SES, Glasgow*		
Communication with other HCPs	Resolved communication problems and disagreements patients had with e.g. Pharmacists, GPs, Respiratory Consultants.	Mainly low SES patients.	*“…I was in and out the hospital all the time…Because they kept changing my medication and changing inhalers. And obviously, they must write to your doctor, and tell them…so please put that on your repeat prescription…but every time I went to the chemist, I was getting the old stuff instead of the new stuff…[Pharmacist] got it all sorted for me, the last time she was out…she went back, and kind of phoned them all and sent them emails and I’ve got it all sorted now, so I get the right medication now.” Participant 5, Female, Low SES, Lothian*	In contact with Respiratory Consultant and GP practice.	*“So, it is not me going in and fixing everything. It is…about me going in and trying to determine the need to the patient and signposting them appropriately as well.”*
	Resolved issues in obtaining appointments.	Low SES patients.		Referrals to other Health Professionals/services.	*“We use quite a lot of probably the rehab teams and dieticians…and quite a lot of the patients have been referred to secondary care for scans of their bones, so for DEXA scanning.”*
	In contact with Respiratory Consultant		*“But [Pharmacist] kept coming for the visit, and she takes my blood pressure and what-have-you and everything. And she relays that to the respiratory consultant…and updates my records. Because everybody that sees it can see the records have been updated.” Participant 8, Male, High SES, Lothian*		
Other health conditions	Diagnosed from tests/scans instigated by Pharmacist.	Mentioned by more low SES patients (but small number)	*“I’ve never had anything done about my heart, so she suggested to the doctor...and he phoned me up, he says, right I’ve had the pharmacist on because of this…so I’ll send you a prescription for statins, take one in the morning. And that was at her suggestion.” Participant 17, Male, Low SES, Glasgow*	Monitored and managed other health conditions.	*“So, you know, we’ve started a lady on hypertension medicines, and we’ve diagnosed a diabetic”*
	Medications and treatments: referred or prescribed			New health conditions diagnosed as a result of tests and scans.	
			*“…actually I was never on a bone, a bone tablet or anything for the...she got me sent for the bone [density]…I hadnae been for one of these for years…and as I say that is when they found out I had all this osteo on the hips and whatever…She put in to [Respiratory Consultant] to get me sent for one and this is when obviously they found that I had a fracture and whatever…So I am on a…tablet every day now but [Pharmacist] had actually put in to [Respiratory Consultant] for to get me on the, it’s an injection. You get it just once a year.” Participant 18, Female, Low SES, Glasgow*	Immunisation uptake monitored.	

COPD – Chronic Obstructive Pulmonary Disease; GP – General Practitioner; HCP – Health Care Professional; OT – Occupational Therapy; SES – Socioeconomic Status.

#### Operationalization of intervention – Trust in Pharmacists (Collective action).

Patients expressed trust in Pharmacists based on their perceived professionalism, experience and knowledge – they described trusting Pharmacists “*100%*” and “*wholeheartedly.*” One patient did express scepticism relating to Pharmacist advice on COVID-19 vaccination. Trust may have been fostered by patients’ perceptions of the advantages of the care provided by Pharmacists and the relationships they developed with them.

#### Operationalization of intervention – Relationship with Pharmacists (Collective action).

Pharmacists were perceived as easy to talk to and good listeners. Patients felt free to disclose information, share worries, concerns and grievances. Pharmacists, patients and their families, built beneficial relationships – enabling pharmacists to ‘read’ patients. For two patients, the Pharmacist was like a friend (Table 3).

#### Evaluation of intervention – Positive impact (Reflexive monitoring).

Most patients had a wholly positive perception of the intervention – one stated participation was “*one of the best decisions I have made in my life”* (Participant 18, Female, Low SES, Glasgow). They appreciated the privacy and comfort of seeing Pharmacists in their home. This regular, accessible support offered reassurance in the context of long waits between appointments and reduced, particularly face-to-face, contact with other HCPs, such as GPs, due to the COVID-19 pandemic. They also gained access to services they did not know about.

Patients appreciated the holistic care provided by Pharmacists and described changes in outcomes. Several patients felt their breathlessness and exacerbations were better managed and issues picked up earlier, faster access to medications (rescue packs [steroid and/or antibiotic medication to treat flare-up of COPD symptoms, as per management plan], new medications, changes to existing medications) and more efficacious medication use. Three patients felt the intervention had contributed to a reduction in the frequency of hospital admissions, others noting a reduction in infections and exacerbations (frequency and/or severity). Several patients were diagnosed and treated for new health conditions (e.g. high blood pressure, high cholesterol). Improved mobility and ability to complete daily tasks following receipt of mobility aids and home adaptations were described by many patients. Two patients reported lifestyle changes – reduced alcohol consumption and improved weight. Pharmacists had also resolved prescription and appointment issues.

Most patients stated they would appreciate continued access to Pharmacist visits, feeling they had been beneficial and reassuring (Table 3).

#### Evaluation of intervention – Limited impact on health (Reflexive monitoring).

Some patients while appreciating Pharmacists visits, expressed a perception that the intervention had not greatly impacted their health. For example, they had not experienced major issues with their COPD/health or felt they were adequately managing their health themselves. However, these patients did report medication changes (some ineffective), investigations (one was diagnosed with a new health condition) and had received mobility aids and rescue packs because of Pharmacist input.

Some patients also noted similarities in care or felt the intervention had reinforced what they had already learned, from their respiratory team or GPs, however the consistency of advice across HCPs is reassuring for the intervention.

One patient felt they would not have benefited from the intervention to the same extent without the disruption in their care caused by the COVID-19 pandemic (Table 3).

#### Evaluation of intervention – Repetitiveness in Pharmacist visits (Reflexive monitoring).

Two patients referred to some repetitiveness in Pharmacist visits, which one felt caused awkwardness (Table 3).

#### Evaluation of intervention – Increased and continued contact (Reflexive monitoring).

Two patients would have liked to have seen the Pharmacists more often, for longer or at an earlier stage of their diagnosis. Having a direct method of contact or leaving a message for Pharmacists between visits would have been appreciated by two patients. At the end of the intervention, Pharmacists and Researchers encouraged patients to ensure they had a note of important numbers/contacts or had asked final questions. However, two participants expressed concerns about losing Pharmacist support, with one feeling “*thrown to the wayside*” (Participant 20, Female, High SES, Lothian) following the end of their visits and would have preferred ongoing support.

#### SES comparison.

Most patients spoke positively of their experience regardless of SES. However, based on patient reports of actions undertaken, it appeared Pharmacists were able to do more for low SES patients. While most patients mentioned being asked about their mental health, high SES patients were more likely to discuss being referred or self-referring for mental health support such as counselling. Low SES patients were more likely to describe issues and delays around diagnosis which may have contributed to their recounting more instances of Pharmacist assistance. While appreciating the intervention, three low SES patients felt it had not greatly impacted their health (Table 4).

### Stakeholder perception of intervention

#### Stakeholder understanding and perception of COPD care and intervention - Professional background/prior experience of HCPs (Coherence).

Pharmacists had prior COPD knowledge and experience through work in primary care and with community respiratory teams. Researchers were experienced in recruitment, home health assessments and pharmacy-related roles which one stakeholder found helped “*enormously*.”

#### Stakeholder understanding and perceptions of COPD care and intervention – Existing health care provision (Cognitive participation).

Access to healthcare services and disruption caused by the COVID-19 pandemic differed within and between geographic areas (e.g. community respiratory teams limited to certain areas). While some patients had continued to receive some form of care during the pandemic, others had little contact with HCPs, increasing a sense of isolation. There was therefore variability in the circumstances and needs of participants in this study (Table 5).

**Table 5:** Stakeholder perception of intervention.

Understanding and perceptions of COPD care and intervention
**Existing health care provision:**
*“Some people…they’ve said that they’ve had great care, people coming out and seeing them, and different teams of people…there’s a few patients…in the same area, who haven’t had any contact from anyone.” Stakeholder 2.*
**Home visits:**
*“…I’m not sure that you would have had attendance…if you’d run the appointments at a GP surgery…And secondly I think it would have been much harder for certain patients to get out to come visit you.” Stakeholder 6.*
**Operationalisation of intervention**
**Good working relationships:**
*“It’s a really great team…and everyone’s involved and works really, really well as a team. So yeah, we’ve got great people on board.” Stakeholder 5.*
**Patients’ health care seeking during trial:**
*“…even just having [Pharmacist] on the end of a phone, is a great help. So, they don’t need to then try and phone the GP to get an appointment…you know, go through all that hassle…” Stakeholder 3.*
**Intervention workload:**
*“It is very challenging when…seeing the new patients because you pull together a lot of information from those initial visits... as you’re trying to wade through hospital notes and get a picture for your patient. I think that was one of the most challenging aspects.” Stakeholder*
**Lone working and lack of workspace:**
*“…because you are on your own and that was another difficult thing with the project, you didn’t have a team to go back and speak to, especially when you’d had a really challenging consultation with a patient and you could just have been doing with having a chat through and kind of processing all the information that you’ve had…” Stakeholder*
**Evaluation of intervention**
**Improved patient health and self-management:**
*“…there’s patients who’ve not been better for years who are much more stable…” Stakeholder 9.*
**Improved patient care:**
*“…some of the non-pharmacological things have…probably helped as well, identifying other problems which maybe were not known about as well…They probably have seen patients who have had very limited contact with GP… or with secondary care, so they have been filling a gap there. Particularly in the aftermath of the COVID pandemic.” Stakeholder 4.*
**Workload of other HCPs:**
*“So, it probably saved the clinician lots of time, but it still takes up a bit of clinician time.” Stakeholder 4.*
**Home visits:**
*“So, I suppose you’ve got an opportunity when you go into someone’s house to observe areas where they might be struggling, so there’s an advantage there. As opposed to a clinic visit or when the patient’s in hospital. It’s difficult to imagine the challenges that might happen in their own house.” Stakeholder 4.*
**Study role rewarding:**
*“Being able to make people improve and to see a difference in people is, just amazing and to see people engaging with other services…And that is hugely rewarding and I know that if I hadn’t been going in to see the patient then maybe we wouldn’t have had the opportunity to engage with that service that I knew about.” Stakeholder*
**Perceived advantage of Pharmacist in this role:**
*“So, I think bringing Pharmacists into COPD management is very much sort of required…they’re certainly a group of patients who would benefit from pharmacy involvement.” Stakeholder 10.*
**Preparing patients for the end of Pharmacist visits:**
*“But I think more will be, as we go along, kind of, in the next sort of six to 18 months to see people if they have regressed. I think that’s going to be more…more difficult.” Stakeholder*

#### Stakeholder understanding and perceptions of COPD care and intervention - Expectations of role and participating in study (Cognitive participation).

Respiratory Consultants were motivated by previous research involvement and the potential impacts of the intervention, although one Consultant expressed uncertainty about the time commitment involved. One Pharmacist highlighted unanticipated aspects of their role such as referrals for oxygen and long-term antibiotics, discussion of alcohol issues, and the extent of their contact with other HCPs.

#### Stakeholder understanding and perceptions of COPD care and intervention - Home visits (Cognitive participation).

The study was designed to provide home visits. Indeed, it was clear from stakeholders’ experiences that home visits had enabled the participation of patients unable to attend other locations and in the context of post-pandemic reduced (face-to-face) contact with HCPs (Table 5).

#### Operationalisation of intervention – Pharmacist tasks and interventions (Collective action).

Pharmacists visited patients at home throughout the intervention and reported undertaking initial assessments, offering patients information and using goal setting techniques, as well as recording interventions, reviewing and making entries in patient’s medical records, and making referrals to other HCPs (Table 4).

#### Operationalization of intervention – Good working relationships (Collective action).

Good working relationships were experienced between HCPs and study staff who were perceived to be “*on board*” and supportive – trust was fostered through past work and collaboration.

The Pharmacist role was “*not a one-person role*” (Stakeholder 6), they were a “*middle person between the patient and consultant*” (Stakeholder 6). Pharmacists also engaged with GP practices to inform them of medication changes, request tests and appointments and discuss hospital admission. Other HCPs/services involved included: Dieticians; Community Link Workers and Respiratory Teams; Pulmonary Rehabilitation; Occupational Therapy; and Mental Health teams. Previous work experience had also equipped Pharmacists with points of contact within NHS services (Table 5).

#### Operationalization of intervention – Patients’ health care seeking during trial (Collective action).

Two stakeholders felt some patients viewed Pharmacists as a substitute to their usual care, instead of an addition (Table 5).

#### Operationalization of intervention – Intervention workload (Collective action).

Pharmacists described significant clinical workload and administrative burdens. The bulk of time-consuming tasks were carried out during initial visits. Beyond this Pharmacists managed changes in patients’ health or life circumstances. Part-time hours and the level of ‘unwellness’ among patients compounded workload challenges.

Due to time pressures, Pharmacists prioritised visiting the most unwell patients and following up others by phone. A Consultant also reported that the intervention generated some additional workload, which could be challenging to incorporate within their usual tasks while ensuring timely responses to Pharmacist requests (Table 5).

#### Operationalization of intervention – Lone working and lack of workspace (Collective action).

Pharmacists’ contact with Respiratory Consultants or other HCPs while frequent, was not face-to-face. A Pharmacist reflected they did not have a team to discuss challenging consultations with or to gain ideas from. Lack of a dedicated workspace in the local NHS was also a challenge (Table 5).

#### Evaluation of intervention – Improved patient health and self-management (Reflexive monitoring).

The intervention was perceived to have improved the health and quality of life of patients. Stakeholders described patients’ reduced exacerbations, improved breathing, and diagnosis/management of other health conditions. Long term input was required to improve some aspects of patients’ health (Table 5).

#### Evaluation of intervention – Improved patient care (Reflexive monitoring).

Pharmacists delivered additional, holistic care to patients and were able to spend more time with them. Pharmacists organised investigations, treatments and referrals which may not otherwise have occurred. There was a perception that patients were receiving safer care and Pharmacist input was valued by other HCPs (Table 5).

#### Evaluation of intervention – Workload of other HCPs (Reflexive monitoring).

A Consultant felt “*quite redundant*” seeing some intervention patients. Pharmacists had carried out many necessary treatment and monitoring tasks e.g. referral for bone density tests. Despite saving Consultants’ time, around an hour of Consultant time per week was required to support Pharmacists (Table 5).

#### Evaluation of intervention – Home visits (Reflexive monitoring).

Home visits were seen to provide better insight and a fuller understanding of patients who were perceived to be more open in their own home, with relatives providing supplemental information. New issues were identified and acted upon quickly, which may not have been picked up in other healthcare settings (Table 5).

#### Evaluation of intervention – Study role rewarding (Reflexive monitoring).

The Pharmacist role was viewed as rewarding, involving relationship building with patients, providing support, implementing changes, and observing improvements. Positive feedback had also been received from patients (Table 5).

#### Evaluation of intervention – Perceived advantage of Pharmacists in this role (Reflexive monitoring).

Pharmacists “*unique skill set*” (Stakeholder 7) and wider expertise was viewed favourably compared to the more specialised COPD/respiratory knowledge of Respiratory Nurses and Physiotherapists. Their ability to prescribe, change and deprescribe medications for multiple health conditions and improve patient understanding was viewed positively. Pharmacists in general were viewed as “*methodical*” (Stakeholder 1) and “*highly skilled*” (Stakeholder 4) (Table 5).

#### Intervention challenges – Perceptions of health and care of patients (Reflexive monitoring).

The needs of patients were perceived as complex. Some were house- or bed-bound, many had difficulties managing at home and were experiencing isolation and loneliness. Pharmacists were challenged by the changeable nature of patients’ health, encountering circumstances which required them to work to the top of their licence. This included decisions around medications, hospital admissions and mental health issues. COVID disruption may have limited training in one site. Despite training and prior respiratory experience, Pharmacists reported encountering respiratory and other clinical scenarios for which they felt further training would have been beneficial and reassuring. A small number of patients were felt to be less receptive to Pharmacists’ input, while less could be done for some who felt they were already adequately managed without the need for additional intervention.

#### Intervention challenges – Preparing patients for the end of Pharmacist visits (Reflexive monitoring).

When the intervention period ended, patients continued to receive usual care. Patients were encouraged to ensure they had asked Pharmacists final questions and kept notes of contacts. However, concerns were expressed regarding the potential deterioration in patients’ health beyond the end of Pharmacists’ visits (Table 5).

### Patient and stakeholder acceptability of trial procedures

#### Patient acceptability – Understanding of study information and intervention (Coherence).

Study information was generally understood. However, some patients noted areas of uncertainty, e.g., exactly what Pharmacists would do.

#### Patient acceptability – Perception of study invitation and support from others to participate (Cognitive participation).

Most patients reacted positively to the study invitation, facilitated for some by a prior conversation with their Respiratory Consultant. Conversations with a study Researcher and a patient’s partner overcame initial reservations of two patients; the families of other patients were supportive of their participation. Patients were motivated to participate in the hopes of benefiting from Pharmacist input, or to help others with COPD and also their Respiratory Consultant.

*“It was [Respiratory Consultant] that actually asked me if I would mind taking part in it…when we were on the phone…and I says that I wouldn’t mind that, so he passed my name and then…got in contact with me.” Participant 16, Female, Low SES, Glasgow.*

#### Patient acceptability – Data collection and researcher home visits (Collective action).

Data collection procedures were generally acceptable, possibly facilitated by the positive interactions and relationship several patients reported with study Researchers. However, trial questionnaires were noted to be long and repetitive, while some questions were not felt to be relevant to a few patients.

*“It’s a repetitive procedure in that they have to see if there’s any change in answer to the questions that they give you…So that’s why it has to be repetitive. But I can understand that. There’s no problem with that.” Participant 10, Male, High SES, Glasgow.*

#### Patient acceptability – Participation in future trial (Reflexive monitoring).

Most patients were willing to participate in a future trial. A small number qualified this decision, depending on trial requirements (time, travel) and their own health, i.e., if experiencing issues that required support.

*“…it’s as if I don’t want to waste anybody’s time. Whereas if I’ve got issues at the time…but then again, you don’t know when you’re going to have the issues. That’s the point. So, you can’t actually say that.” Participant 16, Female, Low SES, Glasgow.*

#### Stakeholder acceptability – Trial patient and staff recruitment (Cognitive participation).

Postal invitations were sent to lists of eligible patients who had moderate or severe COPD and were on a Respiratory Consultant’s outpatient clinic list. Follow-up telephone calls were planned and found to be necessary due to a limited response to postal invitations. Respiratory Consultant endorsement was perceived to have encouraged and motivated some patients’ participation.

*“… it was a lot easier, because the Consultant had already said, we’re doing a study, somebody might be in touch with you. So, it was almost like, you know, there was a foot in the door.” Stakeholder 2.*

However, this approach was not always possible, for example due to staff recruitment delays and COVID impacts. Building relationships with secondary care staff was perceived as important in identifying further patients. Most patients contacted were receptive towards participating in the trial. Reasons for non-participation included: not wishing to be involved in research; previous research participation; being too unwell or not unwell enough; and negative family input.

#### Stakeholder acceptability – Data collection and input, complicated by IT infrastructure (Collective action).

Researchers spent considerable travel and time completing data collection. Study questionnaires were long, with some repetitiveness and questions requiring explanation. Patients could become fed-up or struggled with respiratory symptoms – as a result, questionnaires were occasionally not completed during a single visit. Completing some follow-up questionnaires by phone was preferred by some patients.

*“…you go and see somebody that can hardly breathe, or they’re coughing, a lot of them cough, a lot. So, they maybe have a coughing fit that takes them a good five, ten minutes to recover.” Stakeholder.*

Physical health measures were also collected. However, patients were at times too unwell to complete them, while some also refused to be weighed. Measures from medical records were instead utilised however, these were often outdated.

*“There’s times when I’ve not done [physical measures]. If somebody is so, like mentally unwell, physically unwell, I’m not going to move them…especially if I’m on my own…So I always just document it, you know, not appropriate to move the patient today, or we’d use the last readings we’ve had.” Stakeholder.*

IT access issues working across two sites also caused significant problems at baseline, resulting in a more challenging process for data input which only one person could work on at a time. Data input was time consuming, and use of multiple databases contributed to duplication.

*“…just inputting this information…into the spreadsheets…I think is just taking a lot longer…than they initially thought, because there’s a lot more information that we needed to find that’s not on the questionnaire that we have to go hunting for…I think it’s taken nearly six months to get that all in.” Stakeholder 3.*

However, direct contact with GP practice staff extracting data from patient records was beneficial.

While good safety measures were in place for lone working, intervention and trial staff were often unaware who else would be present in patients’ homes.

Table 6 summarises the key strengths and challenges identified in the pilot RCT from patient and stakeholder perspectives, and their implications for planning a future trial.

**Table 6:** Key strengths, challenges and recommendations.

Key strengths	Recommendations
Professional background/prior experience of HCPs/researchers was found to be beneficial in completing their roles (Stakeholders).	Ideally recruit Pharmacists with respiratory or other relevant background.
Study information was understandable, although some uncertainty of what exactly the Pharmacists could do (Patients).	Study information understandable to patients, could give more examples of support Pharmacists may be able to offer.
Consultant endorsement during recruitment had been a motivating factor for the participation of some patients and had prepared patients for being contacted by Researchers (Patients/Stakeholders).	Input from Respiratory Consultants (speaking to potential participants about the study during consultations) may aid recruitment.
Trust and good relationships between patients, Pharmacists and Researchers facilitated the intervention and trial (Patients/Stakeholders).	Continuation of existing approach to working with patients and between stakeholders.
Home visits appreciated and thought to be advantageous by both patients and stakeholders (Patients/Stakeholders).	Maintain home visits as in the pilot trial, tailored according to Pharmacist assessment of patient needs.
Patients’ and stakeholders’ perceptions of patient health and self-management improved as result of intervention and patients perceived they received additional, safe care (Patients/Stakeholders).	Confirms patient confidence in the intervention and endorses the approaches taken in the pilot trial.
Pharmacists perceived as appropriate to deliver intervention due to knowledge and skills (Patients/Stakeholders).	Reinforces the viability of the approach undertaken in the pilot and confirms that progress to full scale trial is warranted.
**Key challenges**	**Recommendations**
Limited impact on some patients who hadn’t experienced major issues and repetitiveness in home visits. Some patients would have liked more or less visits from Pharmacists (Patients).	Greater flexibility in terms of number of home visits tailored to patient needs.
Similarities to care from other health services/professionals (Patients) and risk of overlap with existing services (Stakeholders).	While reassuring it suggests that important to ensure awareness of local services prior to deployment of intervention in a geographical area.
Study questionnaires long, with some questions requiring explanation, patients became bored, struggled with breathlessness, coughing (Patients/Stakeholders).	Complete some by phone, revisit questionnaires to see if any abbreviation/reduction in data collection possible and check with patient and public partners.
Some patients would have liked a formal method of contacting Pharmacists between visits (Patients).	Dedicated mode of contact (e.g., phone number).
Concerns about care post intervention (Patients/Stakeholders).	Ensure the final Pharmacist visit/contact has a specific format with written end of intervention information provided.
High administrative workload for Pharmacists with data collection and input also time consuming and lack of workspace (Stakeholders).	Additional Pharmacy Technician or other staff needed to ease Pharmacist and Researcher workload and ensure dedicated workspace even if shared. Simplification of data management systems where possible.
Patient population was complex. Pharmacists encountered situations which were challenging and involved working to the top of their licence (Stakeholders).	Ideally recruit Pharmacists with advanced clinical skills/ experience in respiratory and co-morbidities and ensure access to Multi-Disciplinary Teams (MDTs) or other suitable supports.

### Barriers and facilitators to expansion, future larger scale trial and wider implementation

A range of further potential barriers and facilitators to a future, larger scale trial or implementation of the intervention were identified by stakeholders (Table 7). These related to: recruitment, geographic differences in service provision, clinical risk/training, cost effectiveness/funding, IT infrastructure, intervention flexibility, additional staff and resources, and Multi-Disciplinary Teams (MDTs).

**Table 7:** Barriers and facilitators to expansion, future larger scale trial and wider implementation.

Barrier/Facilitator	Description	Some illustrative quotes
Staff recruitment	Barriers: • Staff recruitment delays • Context of NHS staffing challenges may make recruitment of Pharmacists (particularly if requiring respiratory experience) more challenging • Willingness of Consultants to work with Pharmacists	*“…it would just be recruitment, it would be the old problems, of how long it takes to recruit staff to be able to do it…So it would be recruitment of staff, that would probably be the biggest issue, not the recruitment of the patients.” Stakeholder 2.*
		*“…other people might be more efficient and switched on in what they do as a physician that they don’t feel they need to have a Pharmacist checking the homework…” Stakeholder 9*
	Facilitators: • Payment/funding for Respiratory Consultants • Ensure all staff in place before recruitment begins	*“It was a sort of voluntary thing, so it was a kind of unpaid role…I think you’ll need to fund some clinician time in a larger trial and so on.” Stakeholder 8*
Geographic differences and overlap with existing services	Barriers: • Existing NHS structures • Geographical health care inequities • Geographic distribution of COPD • Communication barriers	*“…the barriers…would be the healthcare inequality across Scotland…different things are provided, even within partnership geographical areas, so often the barrier is just communicating across those silos…” Stakeholder 10.*
	Facilitators: • Identify areas of overlap and opportunities for integration into existing services • Geographic distribution of COPD	*“…of course where services are available in one area and not another…and essentially that can cause a little bit of inequality and post code lottery in terms of what services are available to people and I don’t think it’s necessarily a huge barrier but any service would I think, needs to be integrated clearly within the existing structure.” Stakeholder 1*
		*“…I think just ensuring there isn’t significant overlap em you know with services already in place is probably an important thing to establish.” Stakeholder 1*
Clinical risk and Pharmacist training needs	Barriers: • Potential for Pharmacists to be placed in situations of professional risk	*“...any service would, I think need to be integrated clearly within the existing structure…so that everybody knows where they stand in terms of clinical responsibility…so I think just that clear delineation of roles and who to contact needs to be clearly established with this kind of intervention.” Stakeholder 1.*
	Facilitators: • Clear delineation of roles integrated within *existing* structures • Additional Pharmacist training • Implementation of contingency plans for complex/difficult situations • Employing Pharmacists with respiratory care background. • (See also ‘Integration of Pharmacists into Multi-Disciplinary Teams’)	*“So, I guess it’s potentially a discussion around risk of the prescribers going into people’s homes, particularly with patients who can exacerbate and can be very unwell, and are they supported in terms of clinical decision-making and risk assessment of patients at home.” Stakeholder 10*
		*“I would just say that if any Pharmacist was going into this who hadn’t had respiratory training, they would struggle, they would…So, it is a role that you can train Pharmacists to do, undoubtedly. They can do it, but they would require significant training to go out and do this. I wouldn’t want somebody that hadn’t had good background training going out and doing this because they would flounder with these patients.” Stakeholder*
Cost effectiveness/funding	Barriers: • NHS funding constraints • Challenge of communicating cost effectiveness – value of intervention may be in patient wellbeing rather than common metric like reducing hospital admissions	*“The barriers are that there’s a financial barrier in that there are lots of competing areas which need to be funded…we’re probably heading for a period of time where there may be more restrictions in terms of finances, so that’s challenging.” Stakeholder 4.*
		*“…everything in the past has sort of been looked through the prism of saving hospital admissions… But it’s about more than just saving hospital beds, it’s about the you know wellbeing of patients and that sort of thing, okay. Which may translate into better health outcomes. Well hopefully it does, but it might be difficult to get you know robust evidence of that possibly.” Stakeholder 4*
IT infrastructure	Barrier: • Replication of IT issues experienced in pilot RCT which made the input of research data (including information from clinical records) more challenging, particularly if larger scale over multiple sites	*“…if you try and then incorporate that into a wider area, it was difficult over two sites…through different NHS boards that’s going to be a nightmare. So…it’s better to have things stored locally rather than have it all in one place that you can’t access.” Stakeholder 3.*
		*“And the IT infrastructure, as I say. But then, if it was across different sites, you’d have to, I would definitely say, different teams working on their own site.” Stakeholder 2*
	Facilitator: • Data stored locally • Custom database to simplify and assist in management of large volumes of research *data*	*“It could maybe be better done on a kind of database?” Stakeholder 3*
Flexibility in home visits and data collection (impact on workload)	Facilitators: • Further flexibility – inclusion of both in-person home visits and telephone calls; variation in frequency and number of contacts with patients (dependent on health and needs) • Greater emphasis on goal setting • Procedure for discharging patients	*“So, I think that a blended model of visits, certainly you need that initial visit at least one or two and then after that a blended model of visits and telephone calls.” Stakeholder 7.*
		*“I really don’t think you should be going out to visit patients every single time for an intervention. I think interventions can be done over the phone.” Stakeholder 3*
		*“So, I think you would have to introduce some sort of goal setting into the programme as well because if not there wouldn’t be any finality with it.” Stakeholder 6*
Additional staff and resources	Facilitators: • Additional administrative support (Pharmacy Technicians, Pharmacy Support Workers) to support Pharmacists and data collection. • Consideration of increased hours of employment for Pharmacists • Additional Respiratory Consultants in larger scale trial to support more Pharmacists • Provision of dedicated workspaces for Pharmacists and Researchers • Stronger face-to-face relationship with those extracting patient data for research purposes (e.g. Pharmacy Technicians in GP practices) • Asking for data from GP practices at earlier stage	*“And I think perhaps not just having a Pharmacist working on this but like having a Pharmacy Technician working alongside me who could do some of the background work before the visits…that would have made it more efficient…And writing up notes as well and having some kind of admin support for that. So that would maybe be a Pharmacy Support Worker or something like that I think would improve the service.” Stakeholder.*
		*“Well, obviously, we would need more…admin staff; we would need more Pharmacists and Research Assistants, you know, obviously, because, or just, well, or for the Pharmacists to be working more days…* *If it was a larger study, we’d obviously, naturally, need to work more hours, yeah, yes.” Stakeholder 5*
		*“…so, there has to be a requirement if somebody is undertaking similar type of research that they have a base to work from, that that is pre-arranged…” Stakeholder 7*
		*“…obviously, if you’re needing a lot more information on a lot bigger scale…those relationships I think need to be quite strong. So, even maybe having like face-to-face meets…rather than just through email…”* Stakeholder 3.
Integration of Pharmacists into Multi-Disciplinary Teams (MDT)	Facilitators: • Embed Pharmacists within MDTs – further support for Pharmacists, facilitating contact with other HCPs, reducing workload issues including potential clinical risk • Clear communication with primary care to avoid clinical error and duplication of effort	*“So the trial didn’t involve an MDT but…the kind of usual way I would see anyone engaging with managing COPD patients would likely be through some sort of MDT, you know weekly meeting…So longer term if we had a Pharmacist that would be a sort of key meeting for them.” Stakeholder 4.*
		*“I think the communication with the primary care team and whose doing the prescribing and the prescribing changes you know what exactly each person’s role I think communication is really important just to avoid clinical error and also overlap and unnecessary duplication just that there’s clear communication and delineation of whose doing what...” Stakeholder 1*
		*“…my concern would be if you just had the pharmacy service in isolation, without all the other parts of the MDT, so I think that you’d want to ensure that they were well supported and well linked in with other services that could support patients with COPD.” Stakeholder 10*
Patient recruitment strategies	Facilitator: • Replication of strategy involving Respiratory Consultants endorsing trial • Additional recruitment strategies: attending outpatient clinics, pulmonary rehabilitation courses or exercise classes • Dedicated workspace in all sites to reduce delays in patient recruitment	*“…the referral process…I would rather it all worked like that, knowing that somebody has spoken to the patient recently, and that you know, that they’re receptive to the idea that somebody might phone…That was so much more productive I felt.” Stakeholder 2.*
		*“…patients are going into either the outpatient clinics, or if they’re going to pulmonary function testing, or if they’re going to rehab clinics, so sometimes they go and do exercise classes. We could…do the recruitments there. Then, you’re limited because you’re in somebody else’s space, but you could see more patients at the one time.” Stakeholder 2*
Directory of contacts	Facilitator: • Develop directory of contacts over course of Pharmacists’ work which can be shared with other Pharmacists	*“I suppose as you were doing it more you would have a list of contacts that you’re regularly using so you’d have your own directory. And that would then just be multiplied across if there were lots of people all doing it.” Stakeholder*

Abbreviations: COPD – Chronic Obstructive Pulmonary Disease; GP – General Practice; HCP – Health Care Professional; MDT – Multi Disciplinary Team; NHS – National Health Service; RCT – Randomised Controlled Trial.

## Discussion

The TICC-PCP intervention was well received by patients and stakeholders. Patients appreciated regular, accessible contact with a HCP who was able to assess their COPD and comorbidities, act to resolve problems and support them with their COPD, wider health and needs. Many patients described improvements to their COPD management, medications and mobility and most reported positive perceptions, regardless of SES. Stakeholders felt the intervention had a positive impact on many patients, providing them with additional, holistic care which led to improvements in their COPD/health and potentially reducing the workload of other HCPs. Some patients were bypassing GPs, suggesting there is also the potential for this intervention to reduce pressures on GPs. However, some challenges were encountered in the delivery of the trial and intervention, and there were several suggestions regarding how to mitigate these (Table 6).

This positive response resonates with other home or outreach Pharmacist interventions or medicine reviews [8,10]. The feasibility trial preceding the current pilot RCT, found that intervention participants experienced a lower rate of exacerbation, had fewer respiratory hospitalisations and shorter admissions to hospital [7]. A similar intervention among people experiencing homelessness trialled in a pilot RCT, delayed median time to emergency department visit and hospitalisation and also appeared to improve quality of life [10]. Some of the patients participating in this process evaluation reported an improvement in their breathlessness and exacerbation management. A few also felt there had been a reduction in their frequency of being admitted to hospital.

Previous research [9] had identified a lack of a perceived need for medicines review could be a barrier to implementation. In contrast, most TICC PCP participants were motivated to work with the Pharmacist. Patients in our study had moderate-to-severe COPD rather than mild COPD, which may have contributed to higher levels of motivation and need for support. Our findings also resonate with the positive views of patients and stakeholders in a prescribing Pharmacist outreach intervention among people experiencing homelessness [17].

A feasibility study of home outreach by Liaison Health Workers (LHW) for patients with COPD and psychological comorbidity, similarly reported patients appreciating the holistic, personalised approach to their care [18]. However, in contrast to the LHWs’ patients who did not want continued support, most TICC PCP patients would have preferred continued Pharmacist support beyond the trial end [18]. Interventions and pulmonary rehabilitation courses for patients with COPD have faced barriers to recruitment and attendance due to the ability of patients to travel to intervention locations due to distance, inadequate transport and patients’ health and mobility [19–21]. The home-based nature of the TICC PCP intervention was felt to have facilitated patient participation.

While no RCTs have specifically assessed home-based Pharmacist interventions for patients with COPD, a systematic review [22] identified twelve RCTs of home visit interventions by Pharmacists aimed at people with other health conditions, “*at risk of medication-related problems*”. Most interventions were focused solely on advice and medication support; two included some form of lifestyle advice. The review found no evidence these types of intervention influenced hospitalisation, medication adherence, knowledge, or quality of life. However, none of the studies explored interventions delivered by Pharmacist Independent Prescribers, collaborative Pharmacist-Consultant Respiratory Physician interventions or whether perspectives varied among subgroups of the population such as those of a low SES. A further study found that a home-based Pharmacist intervention targeting medically underserved populations, such as older adults with dementia, in Taiwan could significantly improve disease status, medical knowledge, self-care skills and mitigate drug related problems [23].

In terms of learning for a future large-scale trial (Table 6) it was clear that not all patients required the same level of support. A systematic review and qualitative synthesis have suggested that the complexity and unpredictability of COPD requires services to be flexible and promote continuity [24]. While the TICC PCP intervention was tailored to patient needs, including numbers of visits and actions, further flexibility may assist in the management of Pharmacist and MDT workload and matching the needs of patients. Recruitment of additional staff (e.g. Pharmacy Technicians) may also contribute to alleviating workload pressures. While Pharmacists in this study were autonomous clinicians, trained to prescribe and deprescribe or change any medicine, aligning or incorporating this intervention with MDTs could support Pharmacists dealing with new/complex issues and clinical risk. Recruitment of Pharmacists with respiratory experience may be advantageous, nevertheless provision of training related to respiratory conditions and other issues that may be encountered e.g. mental health, could increase the confidence of Pharmacists in dealing with a complex, frail patient population.

The results of this study should also be considered in the context of the continued consequences of the COVID-19 pandemic. Access to health care and treatments such as pulmonary rehabilitation were more difficult, while face-to-face interaction with HCPs was vastly reduced [25,26]. A sense of isolation from support (health services, family and friends), combined with longer-term changes in health care access may have contributed to the positive patient perspective of this intervention. The pandemic may have increased inequalities in COPD and other chronic respiratory diseases [27], which could contribute to the suggestion that low SES patients self-reported more Pharmacist actions.

### Strengths and limitations

The participation of patients from two settings and with a range of socioeconomic environments and a range of stakeholders are strengths, allowing the inclusion of a variety of viewpoints and insights into the procedures and processes involved. Purposive sampling of patient interviewees ensured a balanced sample in terms of sex, geographical location and SES, which enabled a comparison of perspectives. A further strength was the use of NPT which allowed findings to be more explanatory than descriptive as it is a well-established theory used to understand the implementation of complex interventions and self-management of chronic conditions.

A limitation of this study is the small sample size of participants from one country, which restricts generalisability. As control participants were not interviewed, we have been unable to present their perceptions of participating in the trial. A further limitation relates to the reliance on individual recall of events and Pharmacist interventions. Several patients were unable to remember certain aspects of the trial, details of interventions and changes made by Pharmacists or other HCPs. The timing of interviews varied and those interviewed at an earlier stage may not have appreciated any longer-term impacts of Pharmacist actions, while those interviewed later in the intervention may have forgotten some early actions. Some patients may also have conflated the Pharmacist intervention visits with repeated Researcher visits. Finally, as interviews were carried out by telephone, additional insights from body language, facial expressions and patients’ home environments were not collected.

### Implications for future research

Our findings suggest this intervention was viewed positively by patients and stakeholders and demonstrates Pharmacist prescribers were able to undertake a broad range of activities to help patients with COPD and comorbidities. The intervention may be particularly useful for patients from a low SES and consideration could be given to targeting this subpopulation, although further examination of this issue is required. We have identified likely barriers and facilitators to future implementation of the intervention as a form of routine service delivery and made suggestions for mitigation. Overall, the intervention was very positively received. Trial procedures were acceptable and seem to have good implementation potential. If the pilot trial meets the pre-determined progression criteria, then progress to a full scale RCT is merited.

## Supporting information

S1 File Interview schedules and coding trees. (DOCX)

S2 File Consolidated criteria for reporting qualitative studies (COREQ): 32-item checklist. (DOCX)

## Abbreviations

COPD: Chronic Obstructive Pulmonary Disease
DALY: Disability-Adjusted Life Years
GP: General Practitioner
HCP: Health Care Professional
LHW: Liaison Health Worker
MDT: Multi-Disciplinary Team
NHS: National Health Service
NPT: Normalization Process Theory
RCT: Randomised Control Trial
SES: Socio-Economic Status
SIMD: Scottish Index of Multiple Deprivation
TICC PCP: Tailored Intervention at home for patients with moderate-to-severe COPD and Co-Morbidities by Pharmacists and Consultant Physicians
