# Supplementary material for: Process evaluation of a pragmatic, multicentre pilot Randomised Controlled Trial (RCT) in primary care: Tailored intervention for COPD and Co-morbidities by Pharmacists and Consultant Physicians (TICC PCP)
Source: PLoS One. 2025 Jun 30;20(6):e0326178. doi: 10.1371/journal.pone.0326178 (PMC12208426; doi:10.1371/journal.pone.0326178)
Supplement: S1 File — (DOCX) [file pone.0326178.s001.docx]

**S1 File. Interview schedules and coding trees**

**Patient interview schedule**

**Coherence - what is the understanding of the management of COPD and the roles of different health professionals? What do they think of this pharmacist intervention?**

1. What do you understand by the term COPD and what do you have to do to manage your condition?
   1. Can you tell me about being/how diagnosed? When were you diagnosed?
   2. Can you tell me about the symptoms you experience? Have they changed over time?
   3. How do your other health conditions affect you?
   4. Do your other health conditions affect your COPD?
   5. Does taking your COPD medication or medication for other health conditions cause you any problems?
   6. Have you made any lifestyle changes to help manage your COPD or other conditions? E.g. smoking, diet…
2. Before taking part in the study, anything that helped or hindered you in managing your COPD/health conditions or making lifestyle changes? E.g. friends/family, transport, getting prescriptions, getting appointments/advice from HCPs, employment, benefits/carers, home adaptations
3. Which health professionals helped you manage your COPD?
   1. How well do you think they managed all your health problems?
   2. Did health professionals at your GP practice help you manage your COPD and other health problems?
4. What is your experience of annual COPD reviews? Did you attend one while participating in this study?
   1. Do you have any other appointments or annual reviews for your COPD and other health conditions?
5. Has your contact with and care from the health professionals who help manage your COPD (in your GP practice or at the hospital) been affected by the Covid-19 pandemic? If yes, in what ways?
6. When you read the information about the trial what did you think about having a pharmacist come out and help with the management of your COPD and other health problems?
   1. Have you ever interacted with a pharmacist before?
   2. Did you find the information about the study easy to understand? If no, what would have helped? If yes, is there anything that could have been improved?

**Cognitive participation – how well did you engage with the pharmacist?**

1. Why did you decide to take part in the study? What did you hope to get out of it?
2. How did you feel about having to spend time with the pharmacist?
3. Did others (e.g. family/friends/medical professionals etc) help or hinder your participation in the trial or your use of the pharmacist service? Describe how?
4. Did your views of the pharmacist or the trial change over time?

**Collective action – how useful was the pharmacist intervention?**

1. How useful did you find the pharmacist visits? What aspects of the pharmacist visits did you particularly like or dislike?
2. Did the pharmacist intervention help you manage your COPD or any other conditions or problems? If yes, how? If not, why not?
   1. Prompts: Medications (Rescue pack); COPD exacerbation management; mental health; Home aids or adaptations; other health conditions; communication with other health professionals; new diagnosis; lifestyle changes
   2. Did it fit in with the way you manage your COPD/other health conditions at the moment?
3. Did the pharmacist visits make you more or less likely to contact health professionals or health services? Why?
4. How much did you trust the advice/information provided by the pharmacist? Explain.
5. What did you think about the trial questionnaires you had to complete as part of the trial? Did you think any of them asked about things that are particularly important to you? If yes, which ones, if not what would you have liked to be asked about?

**Reflexive Monitoring – overall what did you think about this trial and the help the pharmacists provided?**

1. What aspects of your health/COPD did the pharmacist affect most (positively/negatively)?
2. Do you think the pharmacist offered you different care than you have received before? If yes, how was it different?
3. What, if anything, would you change about the help you received?
4. If someone asked you whether you would recommend the pharmacist visits what would you tell them and why?
5. If possible would you like to have continued access to the pharmacist? Why?
6. Were there any aspects of being involved in the trial that you found difficult or any procedures that you particularly disliked? If yes, what would you like to see done differently in a future large scale trial?
7. Would you participate in a large scale trial of this pharmacist service if approached again? If not, please explain your answer.

**Stakeholder interview schedule**

**Out with this study, could you tell me your job role?**

**Could you describe what your role has been in this study?**

**Coherence - what do you think are the main challenges to optimal care of those with COPD and the roles of different health professionals? What do they think of this pharmacist intervention?**

1. What do you think are the main challenges to optimal care of those with COPD?
2. Which health professionals are currently primarily involved in helping with care delivery of those with COPD and who else, if anyone, should also be involved?
3. What do you think about having a pharmacist come out and help with the management of COPD patients along with their other health problems?

**Cognitive participation – how well did you engage with the pharmacist?**

1. How motivated did you feel to try and work with the pharmacist?
2. How did you feel about having to spend time with the pharmacist?
3. What things or people helped or hindered your participation in the trial or your working with the pharmacist service? Describe how?
4. Did your views of the pharmacist or the trial change over time?

**Collective action – how useful was the pharmacist intervention?**

1. How useful did you find the pharmacist visits? What aspects of the pharmacist visits did you particularly like or dislike?
2. Did the pharmacist intervention help you better manage patients with COPD? If yes, how? If not, why not? Did it fit in with the way you manage patients with COPD at the moment?
3. How much did you trust the advice/information provided by the pharmacist? Explain.
4. What do you see as the main barriers or facilitators to this type of intervention?

**Reflexive Monitoring – overall what did you think about this trial and the help the pharmacists provided?**

1. What aspects of the intervention did you view most positively? Negatively?
2. What, if anything, would you change about the trial procedures or intervention itself going forward?
3. If someone asked you whether you would recommend the pharmacist visits what would you tell them and why?
4. If possible would you like to have continued access to the pharmacist to help provide care for your patients with COPD? Why?
   1. Have you spoken to participants about the end of the study?
5. Were there any aspects of being involved in the trial that you found difficult or any procedures that you particularly disliked? If yes, what would you like to see done differently in a future large scale trial?
6. Would you participate in a large scale trial of this pharmacist service if approached again? If not, please explain your answer.
7. What do you see as the main barriers or facilitators to a future large scale trial? Or Wide scale deployment of these types of services?

**Patient data coding tree**

| **Intervention** | | |
| --- | --- | --- |
|  | **Understanding and Perception of COPD** | |
|  |  | Understanding of COPD |
|  |  | Diagnosis |
|  |  | Progression of COPD |
|  |  | Impact of COPD |
|  |  | Attitude to having COPD and general health |
|  |  | Multimorbidity |
|  | **Management of COPD** | |
|  |  | Management – HCPs involved |
|  |  | Management – Pharmacological |
|  |  | Management – Non-Pharmacological |
|  |  | Other potential facilitators and barriers to management |
|  |  | Management challenges RCT pharmacist can address |
|  |  | Impact of COVID |
|  | **Pharmacist Compared to Other HCPs** | |
|  |  | Perception of management prior to RCT |
|  |  | Previous Pharmacist Interaction |
|  | **Role of RCT Pharmacist** | |
|  |  | Pharmacist ‘roles’ |
|  |  | Tasks carried out by pharmacist |
|  | **Trust in Pharmacist** | |
|  | **Likes about Pharmacist Intervention** | |
|  |  | Relationship with Pharmacist |
|  |  | Intervention design |
|  |  | Intervention ‘fit’ |
|  | **Overall Perception and Impact of RCT Pharmacist Intervention** | |
|  |  | Impact of RCT Pharmacist Intervention on participants |
|  |  | Overall perception of intervention |
|  |  | Change in perception of pharmacist over time |
|  |  | Recommendation |
|  | **Negative Aspects of Intervention and Considerations for Future** | |
|  |  | Negative aspects of pharmacist intervention |
|  |  | Changes to future intervention (increased and continued contact) |
|  |  | Continued access to pharmacist |
| **Trial Procedures** | | |
|  | **Understanding of study information** | |
|  |  | Easy to understand |
|  |  | Difficult to remember |
|  |  | Uncertainty |
|  | **Perception and motivation to take part in study** | |
|  |  | Motivation |
|  |  | Negative perception |
|  |  | Positive perception |
|  | **Data Collection and Researcher Visits** | |
|  |  | Perception of researcher |
|  |  | Perception of study questionnaire |
|  | **Negative Aspects** | |
|  |  | Nothing |
|  |  | No information since intervention over |
|  |  | Changes to the trial |
|  | **Overall Perception of Trial** | |
|  | **Future Participation** | |
|  |  | Would participate |
|  |  | Would participate, but… |

**Stakeholder data coding tree**

| **Intervention** | | |
| --- | --- | --- |
|  | **Professional background, prior experience** | |
|  |  | Administrator prior experience |
|  |  | Participation in pilot study |
|  |  | Pharmacist prior experience |
|  |  | Researcher prior experience |
|  | **Existing Health Care Provision** | |
|  |  | Challenges of managing COPD |
|  |  | Current structures and roles in COPD care |
|  | **Expectations of role and participating in study** | |
|  |  | Aim of intervention |
|  |  | Consultant expectations |
|  |  | Pharmacist expectations |
|  |  | Researcher expectations |
|  | **Role of Pharmacists in study** | |
|  |  | Administrative, data, notes |
|  |  | Home visits |
|  |  | Initial fact finding, patient assessment |
|  |  | Long term input |
|  |  | More than respiratory medication |
|  |  | Pharmacist interventions |
|  |  | Preparing patients for end of intervention |
|  |  | Recruitment |
|  |  | Relationships with other services and HCPs |
|  | **Role of Consultant in study** | |
|  |  | Development of trial |
|  |  | Recruitment |
|  |  | Respiratory consultant works with study Pharmacist |
|  | **Role of GPs in study** | |
|  |  | Pharmacist interaction with GPs, practices |
|  | **Relationships between study team** | |
|  |  | Good relationships |
|  |  | Perception of others involved in study |
|  | **Patient health care seeking during trial** | |
|  |  | Intervention fits in existing care |
|  |  | Patients in study continue to see consultant and GP |
|  |  | Study participants using pharmacist more than other HCPs |
|  | **Intervention Workload** | |
|  |  | Consultant Workload |
|  |  | Pharmacist Workload |
|  | **Lone Working and Lack of Workspace** | |
|  |  | Lack of workspace |
|  |  | Lone working |
|  | **Positive Aspects of Intervention** | |
|  |  | Consultant workload |
|  |  | Importance of home visits |
|  |  | Patient health and self-management |
|  |  | Patients received better care |
|  |  | Pharmacist doing role versus other HCPs |
|  |  | Pharmacist role rewarding |
|  | **Intervention Challenges** | |
|  |  | Perceptions of health and care of patients |
|  |  | Preparing participants for end of intervention |
| **Trial Procedures** | | |
|  | **Patient and Staff Recruitment** | |
|  |  | Patient Recruitment |
|  |  | Randomisation |
|  |  | Recruitment timeline and delays |
|  | **Data collection and Input** | |
|  |  | Challenges of data collection and input |
|  |  | Data input |
|  |  | GP data collection |
|  |  | Patient data collection |
|  | **Role of Researcher in Study** | |
|  |  | Data collection |
|  |  | Data input |
|  |  | Lone working |
|  |  | Planning time and travel |
|  |  | Preparing participants for no longer seeing Pharmacist |
|  |  | Recruitment |
|  |  | Role limited in being able to help people |
|  |  | Training |
|  |  | Working with others in study team |
|  |  | Working across multiple sites |
|  | **Willingness to work on larger scale trial** | |
| **Barriers and Facilitators for Future or Larger Scale Trial or Implementation** | | |
|  | **Barriers** | |
|  |  | Clinical Risk |
|  |  | Cost Effectiveness. Funding |
|  |  | Geographic difference and service overlap |
|  |  | IT Infrastructure |
|  |  | Patient understanding of pharmacist role |
|  |  | Pharmacists being introduced without study evidence |
|  |  | Staffing |
|  |  | Time challenges, workload and frequency of visits |
|  |  | Travel costs |
|  |  | Working in isolation |
|  | **Facilitators** | |
|  |  | Balance of treatment |
|  |  | Being part of a team, MDT |
|  |  | Changes to questionnaires |
|  |  | Dedicated workspace |
|  |  | Directory of contacts |
|  |  | Geographic differences, existing services |
|  |  | GP data collection – relationships and timing |
|  |  | Intervention flexibility |
|  |  | IT infrastructure |
|  |  | Patient recruitment processes |
|  |  | Staffing |
|  |  | Sufficient budget, finance, resources |
|  |  | Training, Experience |
| **Impact of COVID on study** | | |
|  | **Impact of COVID on patients and their health care** | |
|  |  | Health care during pandemic |
|  |  | Mental health |
|  |  | Physical health |
|  |  | Positive aspect of COVID |
|  |  | COPD patient demographics |
|  | **Impact of COVID on RCT** | |
|  |  | Staff training |
|  |  | PPE |
|  |  | Recruitment |
