# Supplementary material for: Process evaluation of a pragmatic, multicentre pilot Randomised Controlled Trial (RCT) in primary care: Tailored intervention for COPD and Co-morbidities by Pharmacists and Consultant Physicians (TICC PCP)
Source: PLoS One. 2025 Jun 30;20(6):e0326178. doi: 10.1371/journal.pone.0326178 (PMC12208426; doi:10.1371/journal.pone.0326178)
Supplement: S2 File — (DOCX) [file pone.0326178.s002.docx]

**S2 File. Consolidated criteria for reporting qualitative studies (COREQ): 32-item checklist**

| Domain 1: Research team and Reflexivity | | | |
| --- | --- | --- | --- |
| Personal Characteristics | | | |
| 1. | Interviewer/facilitator | Which author/s conducted the interview or focus group? | Methods > Process Evaluation > Data Collection |
| 2. | Credentials | What were the researcher’s credentials? | Methods > Process Evaluation > Data Collection |
| 3. | Occupation | What was their occupation at the time of the study? | Methods > Process Evaluation > Data Collection |
| 4. | Gender | Was the researcher male or female? | Methods > Process Evaluation > Data Collection |
| 5. | Experience and training | What experience or training did the researcher have? | Methods > Process Evaluation > Data Collection |
|  |  |  |  |
| Relationship with participants | | | |
| 6. | Relationship established | Was a relationship established prior to study commencement? | Methods > Process Evaluation > Data Collection |
| 7. | Participant knowledge of the interviewer | What did the participants know about the researcher? E.g. personal goals, reasons for doing the research | Methods > Process Evaluation > Data Collection |
| 8. | Interviewer characteristics | What characteristics were reported about the interviewer/facilitator? E.g. bias, assumptions, reasons and interests in the research topic. | Methods > Process Evaluation > Data Collection |
|  |  |  |  |
| Domain 2: study design | | | |
| Theoretical framework | | | |
| 9. | Methodological orientation and theory | What methodological orientation was stated to underpin the study? E.g. grounded theory, discourse analysis, ethnography, phenomenology, content analysis | Methods > Process Evaluation > Data Analysis |
|  |  |  |  |
| Participant selection | | | |
| 10. | Sampling | How were participants selected? E.g. purposive, convenience, consecutive, snowball | Methods > Process Evaluation > Sampling and recruitment |
| 11. | Method of approach | How were participants approached/ e.g. face to face, telephone, mail, email | Methods > Process Evaluation > Sampling and recruitment |
| 12. | Sample size | How many participants were in the study? | Results; Table 1 Interview Patient Characteristics; Table 2 Interview Stakeholder Characteristics |
| 13. | Non-participation | How many people refused to participate or dropped out? Reasons? | Methods > Process Evaluation > Sampling and recruitment |
|  |  |  |  |
| Setting | | | |
| 14. | Setting of data collection | Where was the data collected? E.g. home, clinic, workplace | Methods > Process Evaluation > Data Collection |
| 15. | Presence of non-participants | Was anyone else present besides the participants and researchers? | Methods > Process Evaluation > Data Collection |
| 16. | Description of sample | What are the important characteristics of the sample? E.g. demographic data, date | Methods > Process Evaluation > Sampling and recruitment and Data Collection.  Results; Table 1 Interview Patient Characteristics; Table 2 Interview Stakeholder Characteristics |
|  |  |  |  |
| Data Collection | | | |
| 17. | Interview guide | Were questions, prompts, guides provided by the authors? Was it pilot tested? | Methods > Process Evaluation > Data Collection. Supplementary material |
| 18. | Repeat interviews | Were repeat interviews carried out? If yes, how many? | Methods > Process Evaluation > Data Collection. |
| 19. | Audio/visual recording | Did the research use audio or visual recording to collect the data? | Methods > Process Evaluation > Data Collection. |
| 20. | Field notes | Were field notes made during and/or after the interview or focus group? | Methods > Process Evaluation > Data Collection. |
| 21. | Duration | What was the duration of the interviews or focus group? | Methods > Process Evaluation > Data Collection. |
| 22. | Data saturation | Was data saturation discussed? | Methods > Process Evaluation > Data Analysis |
| 23. | Transcripts returned? | Were transcripts returned to participants for comment and/or correction? | Methods > Process Evaluation > Data Analysis |
|  |  |  |  |
| Domain 3: Analysis and Findings | | | |
| Data analysis | | | |
| 24. | Number of data coders | How many data coders coded the data? | Methods > Process Evaluation > Data Analysis |
| 25. | Description of the coding tree | Did authors provide a description of the coding tree? | Supplementary File |
| 26. | Derivation of themes | Were themes identified in advance or derived from the data? | Methods > Process Evaluation > Data Analysis |
| 27. | Software | What software, if applicable, was used to manage the data? | Methods > Process Evaluation > Data Analysis |
| 28. | Participant checking | Did participants provide feedback on the findings? | Methods > Process Evaluation > Data Analysis |
|  |  |  |  |
| Reporting | | | |
| 29. | Quotations presented | Were participant quotations presented to illustrate the themes/findings. Was each quotation identified? E.g. participant number | Box 1 – Patient Quotes  Table 3 – Pharmacist Actions  Box 2 – Stakeholder Quotes  Results > Patient and Stakeholder Acceptability of Trial Procedures  Table 5 – Barriers and Facilitators to Future, Larger Scale Trial |
| 30. | Data and findings consistent | Was there consistency between the data presented and the findings? | Results: Box 1 – Patient Quotes; Table 3 – Pharmacist Actions; Box 2 – Stakeholder Quotes; Table 5 – Barriers and Facilitators to Future Larger Scale Trial |
| 31. | Clarity of major themes | Were major themes clearly presented in the findings? | Results |
| 32. | Clarity of minor themes | Is there a description of diverse cases or discussion of minor themes? | Results |
|  |  |  |  |

Allison Tong, Peter Sainsbury, Jonathan Craig, Consolidated criteria for reporting qualitative research (COREQ): a 32-item checklist for interviews and focus groups, International Journal for Quality in Health Care, Volume 19, Issue 6, December 2007, Pages 349–357, <https://doi.org/10.1093/intqhc/mzm042>
